# *Lived Lives *at Fort Dunree: a rural Irish community perspective

**DOI:** 10.12688/wellcomeopenres.15613.2

**Published:** 2021-10-07

**Authors:** Eimear Cleary, Kevin M. Malone, Collete Corry, Anne Sheridan, Cecily C. Kelleher, Abbie Lane, Seamus McGuiness

**Affiliations:** 1 Department of Psychiatry, University College Dublin, Dublin, Co. Dublin, Ireland; 2 National Suicide Research Foundation, Cork, Ireland; 3 Health Service Executive, Letterkenny, Donegal, Ireland; 4 School of Medicine, University College Dublin, Dublin, Ireland; 5 Department of Textiles, Galway Mayo Institute of Technology, Galway, Ireland

**Keywords:** Suicide, Stigma, Science-Arts Community Intervention

## Abstract

**Background:  **Elevated suicide rates have alarmed policy makers and communities. In these circumstances, the value of understanding more about communities and their potential role in suicide intervention is becoming more apparent. This study involved evaluating feedback from individuals with and without previous suicidal thinking who participated in an arts-science rural community-based intervention project around suicide in County Donegal, Ireland ( *Lived Lives *at Fort Dunree).

**Methods:**  A combined quantitative and qualitative questionnaire was used to evaluate individual and community responses to the *Lived Lives* project.

**Results: ** Participants ( *n* = 83), with and without a mental health history and previous suicidal ideation, reported they believed *Lived Lives* could have potential to help suicide-bereaved families, people with mental illness and people with suicidal thinking.  Qualitative results suggested its’ suitability for specific groups affected by suicide.

**Discussion: ** The evaluation of the *Lived Lives* project indicated that supervised, “safe-space” community intervention projects around suicide have inherent value with positive impacts for bereaved individuals and communities, including those who have experienced suicidal feelings. Future research should explore the transferability of these findings to other communities, and at-risk groups.

## Introduction

Interdisciplinary approaches are uncommon in relation to suicide research, and arts-science approaches are rare. *Lived Lives* is a unique, durational arts-science project designed to create new knowledge and understanding around suicide through a series of art works and mediated conversations, adapted to the community in which it takes place. The original research project began in 2006 and combined a psycho-biographical autopsy study (a modified psychological autopsy involving both quantitative and qualitative methods) ( Malone, 2013) together with novel “Visual Arts Autopsy” (VAA) methods ( McGuinness, 2010). This involved the research team travelling around Ireland and interviewing 104 suicide-bereaved families, mostly in their homes, about their lost loved one. The psycho-biographical element of the study used a semi-structured interview approach to record clinical and social data on the deceased individuals as told by their families. The additional VAA element invited families to donate images and other personal belongings of the deceased, which became the Visual Arts Archive, from which artist McGuinness created the *Lived Lives* artworks. With informed consent and permission form the donating families, these artworks went from private (firstly, only the families involved saw the works and gave consent) to public and became a central part of the *Lived Lives* engagement model.

**Figure 1.**
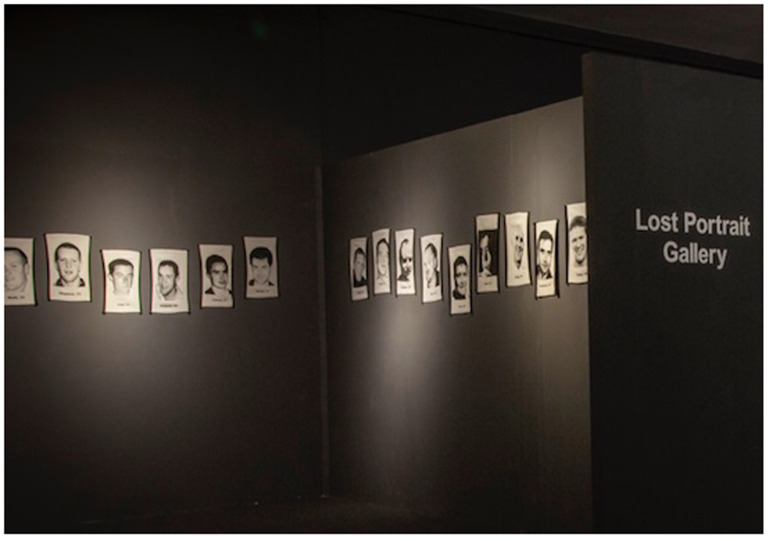
The Lost Portrait Gallery.

**Figure 2.**
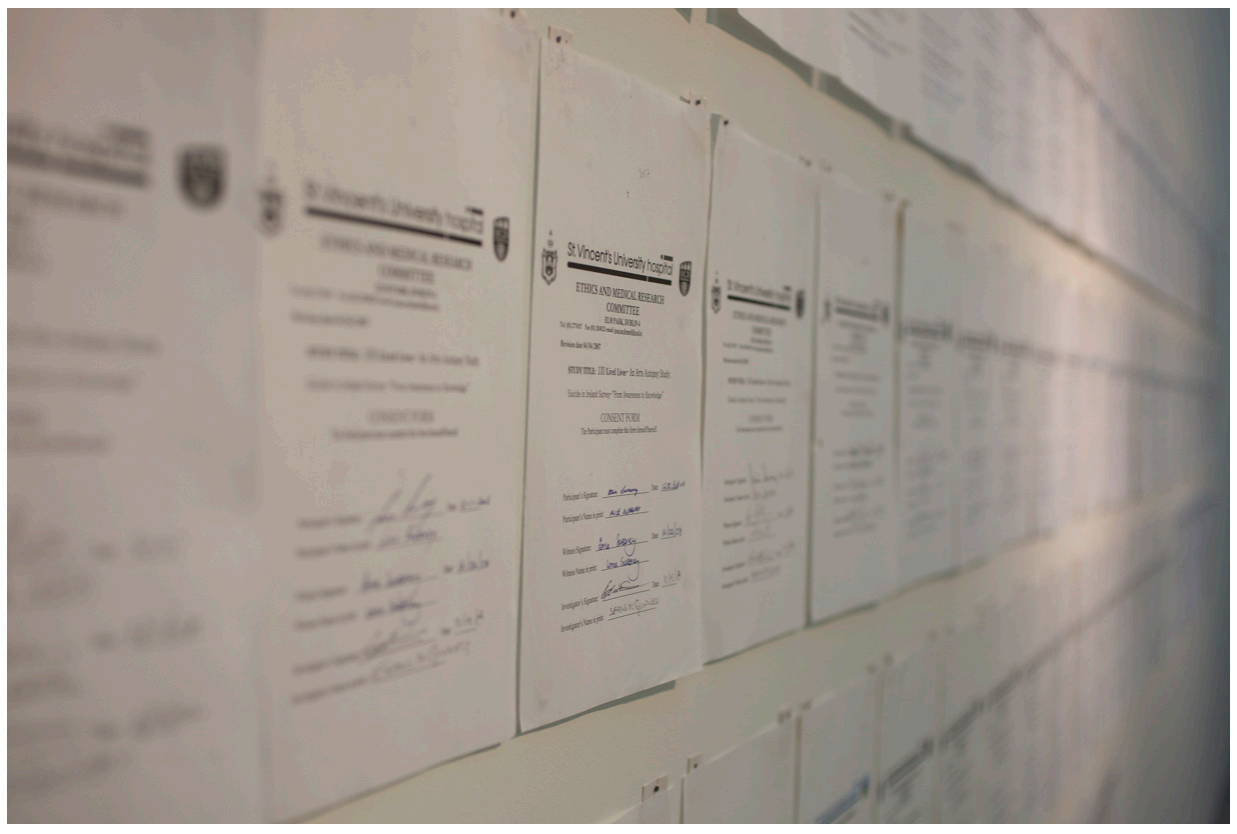
Informed Consent.

**Figure 3.**
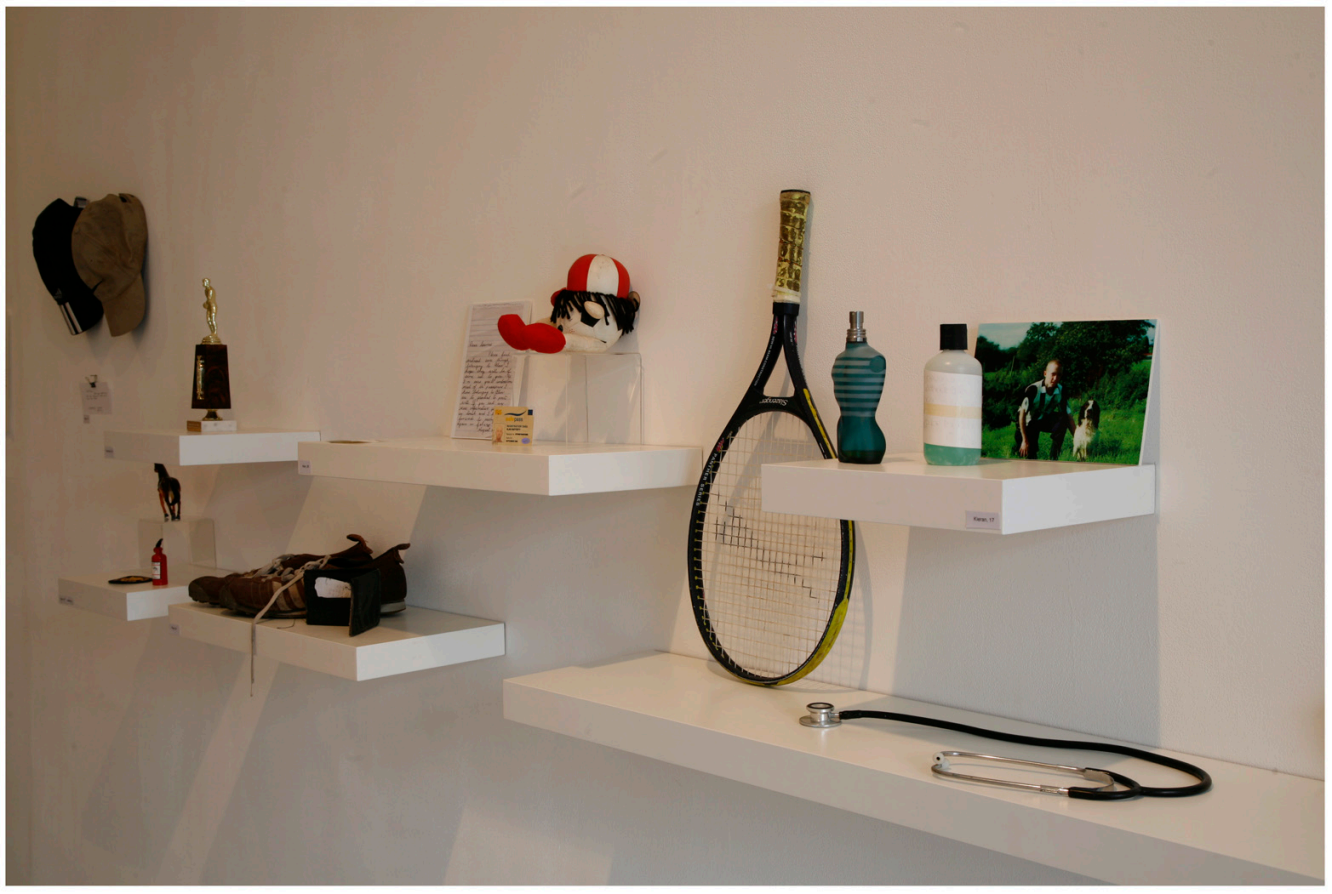
Archive Rooms.

**Figure 4.**
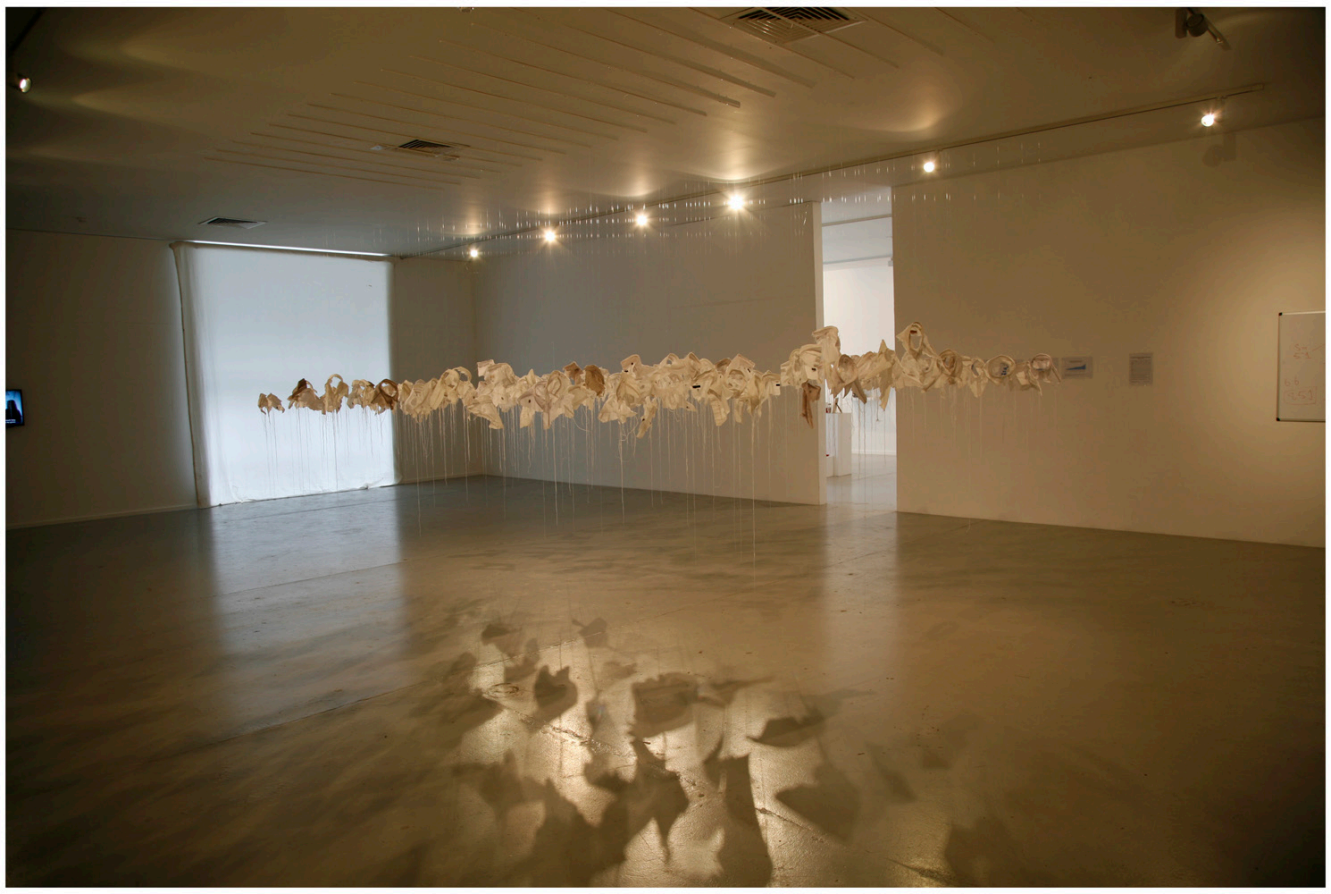
21g.

 The artworks include *The Lost Portrait Gallery*, *Archive Rooms, Informed Consent* and an iteration of *21g* ( McGuinness, 2010). *The Lost Portrait Gallery* (see Figure 1) consists of woven jacquard tapestry portraits of 39 young individuals who died by suicide, worked from donations of photographs and memorial cards donated by the families and friends to the Lived Lives archive. They are a photographic representation in cloth of the deceased. When the artworks are exhibited as the Lost Portrait Gallery, these jacquard tapestries are installed chronologically in a round room according to age at exactly the height of the suicide-deceased individual, unless there is an alternative reason not to. The artwork *Informed Consent* consists of 106 signed consents of *Lived Lives* family participants (see Figure 2). *Archive Rooms* (see Figure 3) consists of clothing, writing and other personal objects belonging to the deceased, donated by the participating suicide-bereaved families, which were originally exhibited as a series of rooms with each loved lost one being represented by their individual belongings. McGuinness originally created *21g* in 2003 as a visual representation of young male suicide in Ireland in that year. *21g* consists of an excess of 92 sculpted fragments of cloth (shirt collars) installed in a group, suspended from invisible threads, one for each young male death that year, and each one weighing exactly 21 grams, the mythical weight of the human soul (see Figure 4).

*21g* is usually adapted to have specific meaning for the community in which *Lived Lives* is taking place. Following the creation of these artworks, a series of site-specific installations and public conversations, mediated by the artist and scientist, took place. These were co-created and presented with the participating research families' consent and involvement. The first engagements of the original research families with the artworks were all documented on video and are now incorporated as part of the *Lived Lives* public engagement model. This involves participants being “walked and talked” through the artworks by the artist, scientist and *Lived Lives* team. As the exhibition is always adapted to the community in which it is taking place, participants are also walked and talked through the suicide statistics most relevant to them and are given information about the issue of suicide in as local a context as possible. Participants are invited to touch and feel the artworks, which facilitate dialogue and response around suicide - “touch the cloth, touch the story” ( McGuinness, 2010). These mediated tours are then usually followed by facilitated round table discussions with the participating group, often amid the physical artworks, where there is an opportunity to discuss their feelings and reactions to the exhibition and their thoughts about the issue of suicide and its impacts. It has been described as a compassionate, consoling and cathartic safe space ( Malone *et al.*, 2015). 

Since its inception, the project has engaged with diverse audiences including suicide bereaved families, school groups, rural community groups and policy makers from the sectors of mental health, arts and culture and public health ( Malone *et al.*, 2015). Most recently, *Lived Lives* was installed in Pavee Point the National Traveller Resource Centre and engaged with the Irish Travelling Community, an ethnic minority in Ireland with significantly increased suicide risk ( Malone *et al.*, 2017). *21g* was recast to reflect the standardised mortality rate (SMR) of 6.6 in male travellers versus the general population. As well as being evaluated by the participants, this week-long exhibition was observed and evaluated by an internal evaluator from the field of visual arts (Janis Jefferies) and an external objective evaluator from the world of suicide research (Christabel Owens) (see Malone *et al.*, 2017). As part of her reflective feedback on *Lived Lives* and what she had observed, Owens described the project as an “intervention” for suicide.

This evaluation and the positive feedback from participants referred to in it, prompted the present study. It raised the question of whether *Lived Lives* could evolve from a community event, engaging and encouraging people to challenge stigma and talk about suicide, to a community intervention, somehow actually having an impact in terms of suicide intervention or prevention for the people who engage with it. Indeed, feedback on earlier *Lived Lives* installations also indicated that people felt there was potential for the project as a suicide prevention initiative. Feedback from public attendees at *Lived Lives* in the Regional Cultural Centre in Letterkenny in 2013 ( Malone *et al.*, 2015) found that 93% of participants believed that the combined art-science model is an effective means for addressing the subject of suicide in Ireland and 80% felt *Lived Lives* could be an effective means of suicide prevention in Donegal.

Community-based approaches are extremely common in public health strategies that attempt to achieve population-level change in risk behaviours and health ( Goodman, 1998). Integral to this approach, according to Merzel & D’Afflinti (2003), is the notion of community participation and ownership in the community-based model for health promotion, as this is considered essential for engagement and generating support within the community the intervention is aimed at. This notion is also central in the *Lived Lives* concept which always expects participating communities to take ownership of the project and be working partners as much as participants (contributors as opposed to consumers). Despite the previous success of community-level population interventions in other areas of public health concern such as smoking cessation and HIV prevention (e,g, Glasgow *et al.*, 1999; Reid *et al.*, 2014) most suicide prevention initiatives to date have focused on change at an individual level. “Finding the Light Within” a public art project which took place in Philadelphia in 2011 and 2012 ( Mohatt *et al.*, 2013) encapsulates a similar community response to suicide. The project involved suicide survivors and individuals bereaved by suicide participating in the design and production of a large public mural about suicide, storytelling and art workshops, and contributing to a storytelling website in an effort to challenge stigma and promote community healing and recovery after suicide. Similarly, the work of Micheal Eales through his “Different Voice, Different Perspective” project (2013) incorporated the personal narratives of those with lived experience of suicide into a series of artworks which aimed to “give voice to the lived experience of suicide from a range of cultural and socio-cultural perspectives” and communicate the impact of suicide on multiple levels. The Lived Lives project also addresses the issue of suicide (and the associated stigma) on multiple levels through a participatory and tactile experience involving art and stories contributed by families bereaved by suicide. Overall however, community approaches such as the “Finding the Light Within”, “Different Voice, Different Perspective” and *Lived Lives* projects are rare when it comes to suicide intervention and prevention.

A recent meta-analysis and systematic review of direct versus indirect psychosocial and behavioural interventions to prevent suicide and suicide attempts by Meerwijk *et al.* (2016) found that interventions that directly address suicidal thoughts and behaviour are effective immediately post-treatment and long term, whereas treatments indirectly addressing these components are only effective long term. “Indirect” in this case referred to treating the symptoms associated with suicidal thoughts and behaviour such as hopelessness or depression. Most of the “direct” intervention studies in the meta-analysis were either dialectical or cognitive behavioural therapy. Indeed, dialectical behaviour therapy has the largest evidence base in relation to long term success in suicide prevention interventions ( Chesin & Stanley, 2013). Elements of *Lived Lives* have potential to be considered a “dialectical” intervention, as there is a logical exploration and discussion of suicide and suicidal feelings involved. Thus, it could also potentially be considered a “direct” intervention for suicide, added to by its community-based approach, which will require further exploration.

Building on this literature about the merits of community and direct interventions, and on previous feedback collected on the *Lived Lives* project, the present study aimed to probe the attitudes to the *Lived Lives* mediated exhibition experience of those within a community with previous experience of mental illness, including suicidal thoughts.

## Methods

### Questionnaire

The study consisted of a semi-structured survey questionnaire relating to the mediated *Lived Lives* arts-science experience ( *Extended data* ( Lived Lives, 2021)). The questionnaire was based on feedback forms used at previous installations ( Malone *et al.*, 2015) and written approval to obtain verbal consent was obtained from St. Vincent’s Healthcare Group Ethics and Medical Research Committee as part of an amendment to the original study (Protocol number 22/17). The questionnaire consisted of six questions in addition to demographic questions. The first three questions were directly related to the participants’ thoughts on *Lived Lives* while the latter three questions were related to their own personal experience of suicide bereavement and mental health history. Question one was solely qualitative while the other five questions allowed participants to tick a “yes”, “no” or “don’t know” box as well as add further qualitative comments if they so wished.

### Procedure

**Figure 5.**
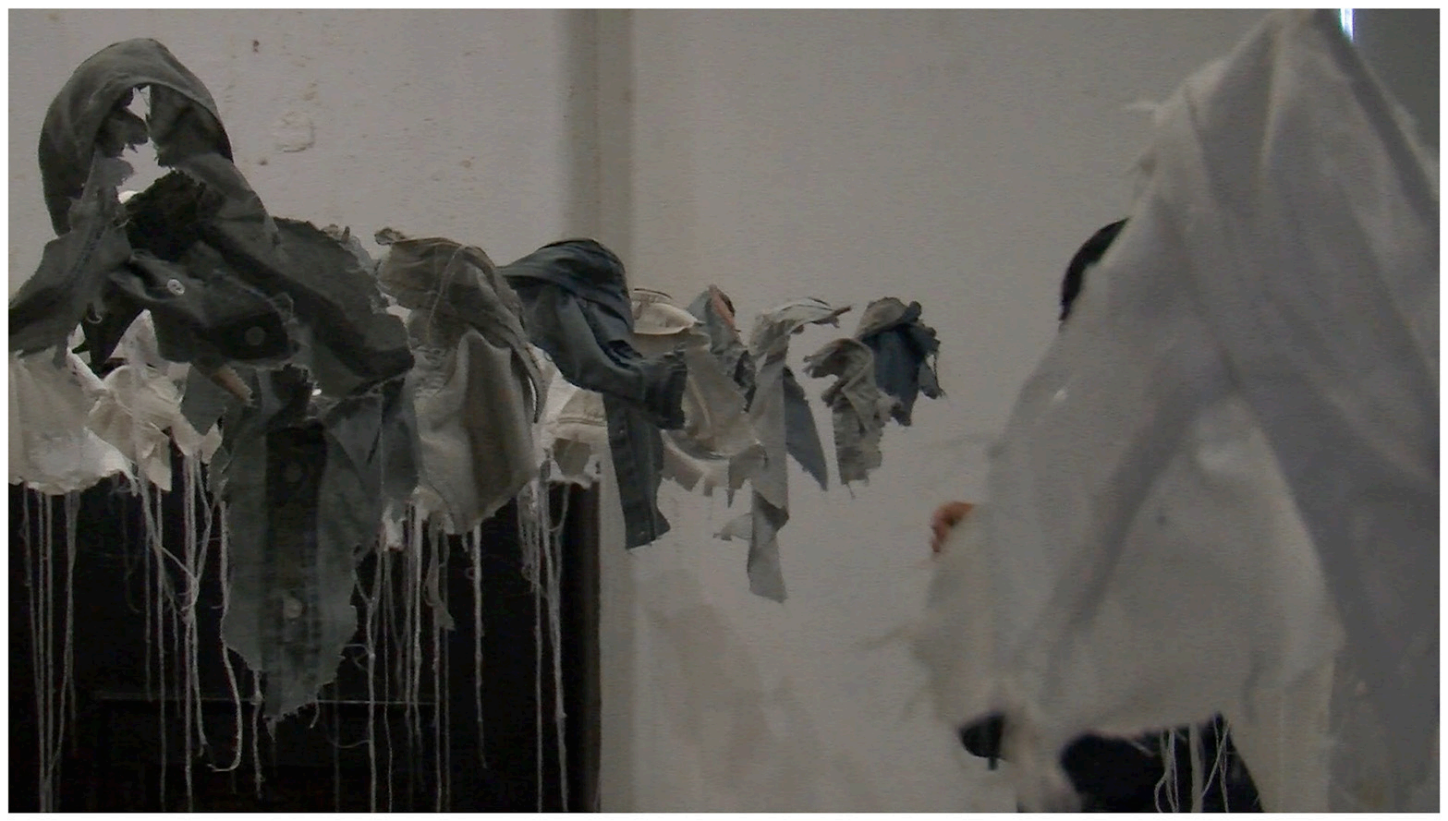
21g Fort Dunree.

Data collection took place over a week-long period in October 2016 at the site of the *Lived Lives* exhibition in Fort Dunree, County Donegal (a free, public event). For this event, the *Lived Lives* artworks were adapted to reflect Donegal suicide deaths over the past decade, including an adapted 21g which was made up of an excess of 156 white and grey shirt fragments reflecting official and unofficial assessments of the number of deaths (see Figure 5). Following a mediated tour of the *Lived Lives* art works and a group discussion of the experience led by the research team of scientist and artist (KMM & SMcG as previously described), participants were given an information sheet and invited to voluntarily participate in the study.. As per the agreed ethical protocol, consent was obtained verbally to protect anonymity. Bereavement support was also present during data collection if any participants wanted to avail of it (none did on this occasion). When completed, participants placed questionnaires in a “ballot box” (to further protect anonymity) and were thanked for their valued time. The anonymous completed questionnaires were stored in a locked cabinet in SVUH Research office. The data from the questionnaires were up-loaded to an encrypted, password protected, dedicated research hard-drive on the hospital mainframe, which was backed up daily,

### Participants

A total of 83 people completed the questionnaire following their engagement with *Lived Lives in Dunree*, ranging in age from 21-71 ( *M*= 46.92, *SD* = 13.15). The sample was 65% female ( *n*= 54) and included everyone that had visited the exhibition over the week and participated in the full mediated experience. Participants included members of the general public who attended as a result of advertising as well as members of local community organisations who were invited to attend. 

### Analyses

Quantitative analysis in the form of frequency analyses and chi square tests was conducted using IBM SPSS Statistics 20. Qualitative analysis was conducted systematically in the form of a step-by-step thematic analysis, as described by Braun & Clarke (2006). A second independent researcher with experience in qualitative analysis also analysed the data (blind to the researcher’s analysis) and identified the same concepts and themes.

## Results

### Quantitative results

Frequency analyses were conducted to assess how many participants answered “yes”, “no” or “I don’t know” in response to the quantitative questions (questions 2–6) across the sample. For questions two and three, the majority of participants answered “yes (75% and 70% respectively). For questions four, five and six which asked participants about their own personal mental health history and whether they had been bereaved by suicide, answers were more evenly distributed between “yes” and “no” with a very small proportion of “I don’t know” answers. Results of all frequency analyses are reported below in Table 1 respectively.

**Table 1.** Frequency analyses.

Question	Response	Frequency	Percentage
***Do you think Lived Lives could benefit someone after a suicide death?***	Yes	62	74.7
	No	3	3.6
	Don’t know	18	21.7
***Do you think Lived Lives could somehow benefit people with mental*** ***health difficulties?***	Yes	58	70
	No	3	4
	Don’t know	22	26
***Have you lost anyone to suicide?***	Yes	37	45
	No	40	48
	Missing data	6	7
***Have you personally ever experienced mental health difficulties?***	Yes	44	53
	No	33	40
	Don’t know	1	1
	Missing data	5	6
***Have you ever experienced suicidal feelings?***	Yes	32	39
	No	45	54
	Missing data	6	7

Chi square analyses were then conducted to assess whether there were any significant between-group differences in these frequencies of “yes”, “no” and “I don’t know” in response to the questions asked. There were no significant age or gender differences among participants who had been bereaved by suicide or had previously experienced mental health difficulties or suicidal thinking themselves versus those who had not. Additionally, the percentage of participants that answered “yes” to whether they thought *Lived Lives* could benefit people following a suicide death (Q2) and whether they thought *Lived Lives* could benefit people with mental health difficulties (Q3) did not differ based on age, gender, previous mental health difficulties or previous suicidal thoughts (all *p*> 0.05). 

### Qualitative results

The four main themes identified in the data were: (1) *Emotive responses to Lived Lives*, (2) *Realising the reality of suicide,* (3) *An insight into the aftermath of a suicide death* and (4) *Is Lived Lives for everyone?* Within theme two, one sub-theme (2.1) of *Raising awareness and challenging stigma* was identified and within theme four there were three sub-themes identified: (4.1) *Live Lives for individuals with mental health issues,* (4.2) *Lived Lives for bereaved families* and (4.3) *Lived Lives for everyone*, *and especially young people*.

***1. Emotive responses to Lived Lives***

The majority of participants used some kind of emotive adjective when describing their experience of engaging with *Lived Lives.* “Sad” and “Sadness” were the most prominent words used as people reflected on what they had heard about the issue of suicide, and seeing those who had died and their families reflected in the artworks:

“Sadness – think these young people felt they had no option but to take their own lives” (Participant (P) 1)

“Sadness, for victims and pity for families” (P48)

Other words used to describe the exhibition and experience overall by participants were “heartbreaking”, “powerful”, “moving”, “touching” and “emotional”:

 “Very emotional. Stark. Real. Heartbreaking” (P67)

“Very powerful and very emotional.” (P8)

Although the majority of participants portrayed that engaging with *Lived Lives* had made them emotional or “moved” them in some way, their comments seemed to suggest that this was not necessarily a negative but a natural part of the effectiveness of the project:

“Sad but not overwhelmed! Privileged to have experienced the exhibition and lucky to have not lost anyone to suicide” (P9)

 “I am overwhelmed by this presentation. I have not cried… I cannot… it is so profoundly sad. It has been to great effect” (P68)

***2. Realising the reality of suicide***

Participants described how engaging with the *Lived Lives* mediated exhibition and the artworks made them realise how real the problem of suicide is in Ireland and how many people are affected annually in their county:

 “It makes it very real – seeing the vacancy their lost life has left behind” (P29)

“It brings it home to you to realise that suicide is very real and can hit any family” (P82)

Many participants also described how seeing the first names, faces, personal belongings and families of individuals who had died by suicide made them think past the statistics they often hear about and think of the actual life and person that has been lost:

“It was human! It gave a human touch to those who died by suicide and those bereaved by suicide. It took away national statistics and replaced the numbers with a person.” (P12)

Although participants described often hearing the statistics about suicide being quoted, being able to touch the artworks and belongings of the lost lives seemed to really bring home this reality and how “normal” people and families can be affected by suicide:

“…We often hear the statistics of these deaths but now to visualise these as actual people again. It shocks us to remember the person. The objects were massively important as I could relate to them in my own life. My sister’s prom dress, my husband’s watch etc., etc.” (P14)

“A very powerful exhibition, thought provoking, really brings home the innocence of lives lost. The belongings of those who died provided a very stark realisation of those who died – the “normality” of their lives. (P65)

One participant (79) described how engaging with *Lived Lives* had given him “a greater appreciation of the fragility of the human mind” while another (P40) reflected “how much it brings home that [ *sic*] could be any of us, we never know what [ *sic*] going on in anyone’s head even when we think we know them inside out”.

***2.1 Raising awareness and challenging stigma***

In addition to many participants expressing how *Lived Lives* had made them realise the reality of suicide, participants also described how they viewed the project as raising awareness by giving them “food for thought” (P26). Participants described how it would cause them to think about the issue of suicide more often or in a new way in the future:

“Emotional – very real, big impact on thinking” (13)

“Really shocking and will think a lot more about who it affects” (P75)

Others related how *Lived Lives* may be challenging stigma around the issue of suicide by giving people a space to initiate conversations and discussions about suicide that does not otherwise exist in Irish society:

““Very powerful, challenging and opened up conversation” (P77)

 “This exhibition allows people to connect and explore all their feelings and the stigmas attached to suicide” (P16)

Engaging with *Lived Lives* also seemed to make some participants feel they “need to do more” (P19) in terms of suicide prevention, both personally and in their professional work, as they described their increased awareness about the issue of suicide causing them to become concerned and scared that the young people in their lives might be at risk.

***3. An insight into the aftermath of suicide***

Participants described watching the videos and hearing the stories of families bereaved by suicide as giving them a new insight into the aftermath of suicide for those left behind and how one death can affect so many, in what one participant (43) described as a “ripple effect”:

“Gives a brief glimpse of the sadness and grief that the families must experience” (P5)

“I think people benefit from seeing what the after is really like” (P16)

Many participants felt that the insight into the grief and pain of families who have lost someone to suicide that *Lived Lives* gives could encourage people who are contemplating suicide to seek help. Perhaps if they saw the potential impact on their families and also what a loss they would be to them, they might be forced to think differently about their situation:

 “Making them aware of the help that is there and the devastation that suicide causes” (P36)

“…it does have the potential to make them think more about the effects of suicide and if anyone was considering suicide as an option I believe they couldn’t help but be moved by what they saw here” (P65)

One participant actually echoed the thoughts of other participants in relation to seeing the impact of suicide on families when sharing her personal experience: “It made me think of the recent times that I have felt suicidal. I am glad I didn’t put my family through that pain and I hope I never do” (P44). Another participant with previous suicidal thoughts (P48) said “at a young age I felt like I had nobody and never thought of my family. But this would make you think”.

As well as impact on families left behind, some participants felt that *Lived Lives* could help those with suicidal feelings also truly think about the finality of suicide for themselves:

“( *Lived Lives*) emphasised the finality of suicide, to seek help” (P32)

“I think it helps people realise dead is dead, there is no coming back” (P16)

However, others questioned whether those in despair would be in the mental state to logically think through the impacts and outcomes of their decision:

“I think most people would be aware of the hurt they would cause if they killed themselves - but when they are in the depths of despair they probably can’t think about other people’s feelings.” (P65)

***4. Is Lived Lives for everyone?***

***4.1 Lived Lives for those with mental health issues***

In addition to discussing how *Lived Lives* may encourage help-seeking through showing the impact and finality of suicide to people, participants also discussed more generally the possible risks and benefits of people with mental health issues viewing the exhibition. In terms of benefits, participants described the potential of *Lived Lives* to make people aware that they aren’t alone; not only in terms of getting help, but in terms of realising other people have experienced similar pain and thoughts to what they may be going through. However, a small number of participants questioned whether the content of the exhibition might be too shocking for those struggling with their mental health or with suicidal feelings:

“I would fear if someone was feeling suicidal it might push them over the edge. Please thread [ *sic*] carefully!” (P29)

“Hope for the future is needed; find that (this) [ *sic*] does not provide this” (P13)

Although the majority of participants (70%) said they felt it could be beneficial in some way, some seemed reluctant to explicitly recommend it to others with mental health issues. One participant (P12) who describes herself as being “suicidal myself a few years ago” said “It depends; everyone interprets things differently. I would find it beneficial but others may not.” There seemed to be a general consensus among the participants that any benefits to those with mental health issues or suicidal feelings would only be seen if they had the adequate supports in place and if the exhibition was done in a sensitive, guided way by experienced professionals:

“… it could be a step forward for people if facilitated sensitively. But Feelings that arise must be given time to be acknowledged, named and facilitated accordingly. How you do it is just as important as what you do.” (P28)

***4.2 Lived Lives for bereaved families***

Similar to the issues raised in the previous theme, participants also discussed the possible risks and benefits of families bereaved by suicide engaging with *Lived Lives.* Many participants named timing and how long it has been since the bereavement as an important factor to consider:

“It is a grieving place, and those people left behind will need time, space to do so” (P21)

“Probable care needed – people need to be ready” (P25)

With the right timing and support, participants felt that *Lived Lives* could be beneficial to suicide bereaved families in a number of ways. Many felt it would be helpful for them to see how many other families have been affected and meet others who had also been bereaved by suicide, to feel they are not alone:

“Communal feeling creates support” (P22)

“I feel like it could give them some reassurance or comfort knowing others have and feel as they do and pain they have and have the same unanswered questions” (P46))

Others felt it could be therapeutic or comforting for bereaved families by allowing them to remember their loved one lost to suicide in a new way. Indeed, some participants directly spoke about how they felt about engaging with *Lived Lives* as someone who had been bereaved by suicide:

 “I entered apprehensive. I am leaving a little lighter for my own loss” (P43)

“Immediate feelings – heartbreak, sick, something sitting on my chest, all the feelings I have come to know with grief. Going around the exhibition brought a sense of calm to see people remembered in a real sense” (P69)

***4.3 Lived Lives for everyone, and especially young people***

Despite the reservations of some participants about who should see the project and when, others felt that it could only have positive impacts in terms of suicide prevention in communities and should reach as many people as possible:

“It should be spread out more through the country on a regular ongoing basis” (P53)

“There needs to be more of this” (P38)

Participants seemed to think young people especially “needed” to engage with the project and would particularly benefit from it. Many suggested working with schools to reach young people who may be a specific risk group for suicide and mental health issues:

“Amazing, all school, youth services should bring young people to see this” (P47)

“…Should be implemented into school trips – young people need to be aware that these decisions are real and families will suffer through anguished decisions – may improve how they feel” (P48).

## Discussion

To the best of our knowledge, no previous study has evaluated the potential of an arts-science community model for suicide intervention, incorporating suicide-bereaved families and within a rural community where some had experienced previous suicidal thoughts. The quantitative results indicate that the vast majority of participants felt that there are potential benefits in the *Lived Lives* project for families bereaved by suicide and for individuals who may be struggling with mental health issues or suicidal ideation themselves. There were very few outright “no” responses to these questions. There were however a small number of “I don’t know” responses, which the space for qualitative responses gave participants a chance to illustrate and explore.

Although suicide figures and statistics are commonly reported and talked about in Ireland, seeing and hearing about these statistics in a more local context (as the exhibition was “personalised” for County Donegal), and also seeing the actual first names and faces of suicide-deceased people on the jacquard tapestries seemed to have a particular impact. This community adaption element, as well as looking at the “lost life” that comes with a suicide death appears to be what led to the “realisation” many participants described coming to about the reality of suicide. Denial is a common factor post-suicide bereavement and a coping mechanism many adapt ( Sudak *et al.*, 2008). The *Lived Lives* experience presents the opposite – it is “real” and directly addresses suicide. The results suggest this direct approach moves participants beyond denial to begin a journey of exploration and discussion. For those with previous suicidal thoughts, perhaps the aftermath and finality of suicide becomes only too apparent. In the case of suicide bereaved families, this direct safe space seems to facilitate mourning, acceptance and catharsis.

This in turn leads to the question of the different psychological processes that might be at play when experiencing and engaging with *Lived Lives*. For people with previous suicidal thinking, witnessing the impact of a suicide death on those left behind may act as the preventative or intervention measure. This type of aversive intervention approach has been extensively used, and with some success, in other public health campaigns such as road safety (e.g. “Crashed Lives” advertising campaign, Road Safety Authority, 2017) and smoking cessation ( Glasgow *et al.*, 1999). There may also be prevention potential in discussing the finality of suicide (especially with youth who may be more inclined to deny mortality) and meeting others who have found themselves in similarly dark places, but “survived”. Although those bereaved by suicide may bear an increased risk for suicide and psychiatric care themselves ( Pitman *et al.*, 2014) as they often experience symptoms of depression and post-traumatic stress disorder after a suicide death, *Lived Lives* may also offer a different type of “intervention”. Being bereaved by suicide can often lead to feelings of rejection and shame in communities ( Pitman *et al.*, 2016). Being able to share stories of loved ones lost, openly discuss the unanswered questions and express emotion in an inviting, open and non-judgemental safe space, created by the *Lived Lives* project and situated in their own community, could be the pivotal moment in starting a therapeutic process for many bereaved. Although local bereavement counsellors were available throughout the exhibition, they we not called upon to provide any acute intervention but provided important background consolation. However, as pointed out by some participants in their qualitative responses, what works for one person, may not work for another, which needs to be borne in mind when interpreting the potential of *Lived Lives* for those with mental health issues ( *explored further in Malone *et al.*, 2019*). Similarly, stages of grief vary for suicide-bereaved families, which makes prior information and informed consent integral to engagement with *Lived Lives*.

Many participants suggested that the *Lived Lives* project would be especially beneficial for young people and schools to participate in as they are a community moving through a period of increased risk. There is a growing evidence base in relation to the effectiveness of school-based suicide intervention initiatives such as the Saving Young Lives in Europe (SEYLE) multicentre trial, which included 168 countries including Ireland. The SEYLE study found that suicide attempts and suicidal ideation in pupils randomly assigned to an intervention group which involved participating in a mental health programme was significantly reduced during a 12 month follow up ( Wasserman *et al.*, 2015). Although the present study focuses on adult respondents, the *Lived Lives* project has engaged with an estimated 800 school students since its inception (see McGuinness & Malone, 2019). Feedback from these engagements suggests that young people find the experience particularly effective as they can relate to the lives of the young people featured. Many students also reported that it caused them to think about the effects of suicide on families, something which many reported they had not contemplated before visiting *Lived Lives*. The full results and feedback from engagement of under 18’s with the project will be the focus of a future publication (see McGuinness & Malone, 2019).

53% of participants stated they had personally experienced mental health difficulties and 39% of participants indicated they had experienced suicidal thoughts in the past. Many studies have found that the majority of people who attempt suicide seek help in some way beforehand, many by disclosing their suicidal thoughts to someone ( Choi *et al.*, 2017) or engaging in increased healthcare contact is often common prior to a suicide attempt with ( Ahmedani *et al*., 2015). Despite this, it is clear a stigma remains in relation to suicide and help seeking. In a survey by St. Patrick’s Mental Health Services (2015) 22% of participants said they would not tell anyone if they were experiencing suicidal thoughts and 72% of participants felt that being treated for a mental health problem is still seen by Irish society as a sign of personal failure. These results may be an insight into the elements that make *Lived Lives* a potentially safe and compassionate space - a site of not only mourning, but of open conversation and acceptance where people feel comfortable enough to share thoughts about what for many is possibly the worst moments of their lives.

 No mixed method study is without its’ limitations. O’Cathain & Thomas (2004) discuss the pros and cons of including the types of open-ended questions used in this study, as the data collected as “comments” on surveys can pose difficulty for researchers in terms of analysis and reporting. However, spaces for free text can also be very beneficial and illustrate of quantitative data. In this case, following “closed” questions with open questions (“any further comments”) allowed the participants to corroborate their answers to the closed questions and voice their opinions – ensuring a power balance between researcher and research participant. As the primary aim of this study was to analyse feedback on the *Lived Lives* project for individuals with experience of suicidal thoughts in particular, these general open questions were included with the intention that participants could elaborate on their general experience, voice their opinions and highlight any issues or concerns freely. However, it is important to note that while the researcher hoped the open-ended style of the questionnaire created the space for any concerns to be addressed, it was not directly asked whether they felt the intervention could have any potential negative impacts which limits interpretation of results. Participants were however directly asked as part of the mediated discussion following the event whether they had any regrets about participating. No participant expressed any regrets in doing so although it must be acknowledged that there may have been potential response bias given the presence of the research team.

The design of the questionnaire had a formatting error in relation to question 6; “Have you ever experienced suicidal feelings?”. There was an option to tick yes or no, followed by “If *yes, do you think the Lived Lives project/exhibition could reduce suicidal feelings?”* However, there was no “yes” or “no” box before the next part which read “ *If so, how?”* and provided a qualitative space Thus, the layout led to one “yes/no” section being left blank. Nonetheless, the information gathered was of interest, bearing in mind this study was the first enquiry about introducing *Lived Lives* to those with previous mental health issues and suicidal thoughts. Future research will hopefully gain a deeper insight into the thoughts of suicidal communities on the project.

One must also acknowledge the limitations of a volunteer sample and a small sample size. The event may have attracted those who were more open and willing to discuss suicide and acknowledge their own feelings on the topic. The public elements of the event took place during working hours and during the week (with the exception of one day) and so those in full time employment or full-time education may have been under-represented. However, the project took place in a very rural community in Ireland, typically hard to reach in terms of research. Although there were more females than males, there were still quite a high proportion of male respondents considering males are traditionally very difficult to recruit for studies on personal topics such as suicide or mental health ( Woodall *et al.*, 2010). Although the data is cross-sectional, this may actually be an integral part of the “intervention” element of *Lived Lives* as the project is adapted and mediated with specific communities in mind and made possible by working in partnership with local organisations on the ground.

The results overall suggest *Lived Lives* is an innovative and progressive cathartic “safe-space” which contains elements consistent with a community suicide intervention. Individuals with and without a history of mental illness and suicidal thinking gave positive feedback about their experience. Having probed the reactions of those with a history of suicidal thinking in a community setting with positive results for the majority, evaluating the project in psychiatric or mental health settings could possibly be a worthwhile and progressive next step in future research of *Lived Lives.* This would permit a fuller assessment of its potential as an intervention within a psychiatric community, for patients and staff alike, and is the focus of Paper II: *Bringing Lived Lives to Swifts Asylum; a Psychiatric Hospital Perspective* ( Malone *et al.*, 2019). How elements of the project may be replicated and used internationally in the context of suicide intervention and prevention is also explored in this paper.

## Data availability

### Underlying data

Irish Social Science Data Archive: Lived Lives, https://www.ucd.ie/issda/data/livedlives/ ( Lived Lives, 2021). Study number (SN): 0070-00

This project contains the following underlying data:

- Lived_Lives_Data_Paper1_Fort_Dunree (Word file containing quantitative and qualitative data collected at Lived Lives Fort Dunree)

These data are under restricted access due to the sensitivity of the subject material. To access the data, please complete a ISSDA Data Request Form for Research Purposes, sign it, and send it to ISSDA by email ( issda@ucd.ie). Researchers will be asked to provide a description for the intended use of the data and will be asked to agree to the terms of use, as outlined in the request form. Data access will be granted for teaching and research purposes under ISSDA terms and conditions.

### Extended data

Irish Social Science Data Archive: Lived Lives, https://www.ucd.ie/issda/data/livedlives/ ( Lived Lives, 2021). Study number (SN): 0070-00

This project contains the following underlying data:

- Lived Lives Fort Dunree Blank Questionnaire (Copy of questionnaire used at Lived Lives Fort Dunree)

Data are available under the terms of the Creative Commons Attribution 4.0 International license (CC-BY 4.0).

## References

[ref-1] Ahmedani BK Stewart C Simon GE : Racial/Ethnic differences in health care visits made before suicide attempt across the United States. *Med Care.* 2015;53(5):430–5. 10.1097/MLR.0000000000000335 25872151 PMC4397662

[ref-2] Braun V Clarke V : Using thematic analysis in psychology. *Qual Res Psychol.* 2006;3(2):77–101. 10.1191/1478088706qp063oa

[ref-4] Chesin MS Stanley BH : Dialectical behaviour therapy for mood disorders.In J. Mann & P. McGrath (Eds). *Clinical Handbook for the Management of Mood Disorders*. New York: Cambridge University Press.2013;280–288. 10.1017/CBO9781139175869.021

[ref-5] Choi NG DiNitto DM Marti CN : Older suicide decedents: intent disclosure, mental and physical health, and suicide means. *Am J Prev Med.* 2017;53(6):772–780. 10.1016/j.amepre.2017.07.021 28985982

[ref-24] Eales MJ : Different Voice, Different Perspective: an arts-based and evocative research response to original voice narratives of suicide. (Doctoral dissertation, Southern Cross University).2013. Reference Source

[ref-6] Glasgow RE Vogt TM Boles SM : Evaluating the public health impact of health promotion interventions: the RE-AIM framework. *Am J Public Health.* 1999;89(9):1322–1327. 10.2105/ajph.89.9.1322 10474547 PMC1508772

[ref-7] Goodman RM : Principals and tools for evaluating community-based prevention and health promotion programs. *J Public Health Manag Pract.* 1998;4(2):37–47. 10.1097/00124784-199803000-00006 10186732

[ref-26] Lived Lives: Extended data. Available at Irish Social Science Data Archive: Lived Lives, Study number (SN): 0070-00.2021. https://www.ucd.ie/issda/data/livedlives/

[ref-8] Malone KM : Suicide in Ireland 2003–2008.Dublin: 3T’s Charity.2013. Reference Source

[ref-9] Malone K McGuinness S Sheridan A : Lived lost lives - a science / arts research collaborative: engaging bereaved families, policy makers and the public with youth and young adult suicide.American Psychiatric Association Annual Meeting, Toronto.2015.

[ref-11] Malone KM Cleary E Kelleher C : Bringing Lived Lives to Swift’s Asylum: A Psychiatric Hospital Perspective [version 1; peer review: 1 approved with reservations]. *Wellcome Open Research.* 2019. 10.12688/wellcomeopenres.15588.1 PMC8980674 35425863

[ref-10] Malone KM McGuinness SG Cleary E : *Lived Lives: a pavee perspective*. an arts-science community intervention around suicide in an indigenous ethnic minority [version 1; peer review: 3 approved]. *Wellcome Open Res.* 2017;2:27. 10.12688/wellcomeopenres.11330.1 28540367 PMC5439511

[ref-12] McGuinness S : Lived Lives.Unpublished PhD Thesis, School of Medicine and Medical Science, University College Dublin.2010. Reference Source

[ref-13] McGuinness S Malone KM : Lived Lives: From Tory Island to Swift’s Asylum.E-publication.2019. Reference Source

[ref-14] Meerwijk EL Parekh A Oquendo MA : Direct versus indirect psychosocial and behavioural interventions to prevent suicide and suicide attempts: a systematic review and meta-analysis. *Lancet Psychiatry.* 2016;3(6):544–554. 10.1016/S2215-0366(16)00064-X 27017086

[ref-15] Merzel C D’Afflitti J : Reconsidering community-based health promotion: promise, performance, and potential. *Am J Public Health.* 2003;93(4):557–574. 10.2105/ajph.93.4.557 12660197 PMC1447790

[ref-16] Mohatt NV Singer JB Evans AC Jr : A community's response to suicide through public art: Stakeholder perspectives from the *Finding the Light Within* project. *Am J Community Psychol.* 2013;52(1–2):197–209. 10.1007/s10464-013-9581-7 23743604 PMC3865777

[ref-17] O'Cathain A Thomas KJ : “Any other comments?" Open questions on questionnaires - a bane or a bonus to research? *BMC Med Res Methodol.* 2004;4(1):25. 10.1186/1471-2288-4-25 15533249 PMC533875

[ref-19] Pitman A Osborn D King M : Effects of suicide bereavement on mental health and suicide risk. *Lancet Psychiatry.* 2014;1(1):86–94. 10.1016/S2215-0366(14)70224-X 26360405

[ref-18] Pitman AL Osborn DPJ Rantell K : The stigma perceived by people bereaved by suicide and other sudden deaths: A cross-sectional UK study of 3432 bereaved adults. *J Psychosom Res.* 2016;87:22–29. 10.1016/j.jpsychores.2016.05.009 27411748 PMC4988532

[ref-20] Reid AE Dovidio JF Ballester E : HIV prevention interventions to reduce sexual risk for African Americans: the influence of community-level stigma and psychological processes. *Soc Sci Med.* 2014;103:118–125. 10.1016/j.socscimed.2013.06.028 24507916 PMC3920181

[ref-21] St. Patrick’s Mental Health Services: Attitudes and awareness survey: summary of key results 2009-2013.Dublin: St. Patrick’s Mental Health Services.2015.

[ref-22] Sudak H Maxim K Carpenter M : Suicide and stigma: a review of the literature and personal reflections. *Acad Psychiatry.* 2008;32(2):136–142. 10.1176/appi.ap.32.2.136 18349334

[ref-23] Wasserman D Hoven CW Wasserman C : School-based suicide prevention programmes: the SEYLE cluster-randomised, controlled trial. *Lancet.* 2015;385(9977):1536–1544. 10.1016/S0140-6736(14)61213-7 25579833

[ref-25] Woodall A Morgan C Sloan C : Barriers to participation in mental health research: are there specific gender, ethnicity and age related barriers? *BMC psychiatry.* 2010;10(1):10. Reference Source 21126334 10.1186/1471-244X-10-103 PMC3016310

