# Supplementary material for: Lived Lives at Fort Dunree: a rural Irish community perspective
Source: Wellcome Open Res. 2021 Oct 7;6:86. Originally published 2021 Apr 19. [Version 2] doi: 10.12688/wellcomeopenres.15613.2 (PMC8548848; doi:10.12688/wellcomeopenres.15613.2)
Supplement: Peer review history

# Peer review: Reviewer response for version 2

**DOI:** 10.21956/wellcomeopenres.19081.r46325

**Published:** 2021-11-03

**Authors:** Hyeouk Chris Hahm

**Affiliations:** 1 School of Social Work, Boston University, Boston, MA, USA

Thank you for the revision. The paper should be accepted.

Is the work clearly and accurately presented and does it cite the current literature?

Yes

If applicable, is the statistical analysis and its interpretation appropriate?

No

Are all the source data underlying the results available to ensure full reproducibility?

Yes

Is the study design appropriate and is the work technically sound?

Yes

Are the conclusions drawn adequately supported by the results?

Yes

Are sufficient details of methods and analysis provided to allow replication by others?

Yes

Reviewer Expertise:

Mental health, suicide prevention.

I confirm that I have read this submission and believe that I have an appropriate level of expertise to confirm that it is of an acceptable scientific standard.

# Peer review: Reviewer response for version 2

**DOI:** 10.21956/wellcomeopenres.19081.r46324

**Published:** 2021-10-26

**Authors:** Erminia Colucci

**Affiliations:** 1 Department of Psychology, Middlesex University, London, UK

I am satisfied with the way the authors have addressed my and the other reviewer’s suggestions thus the status can now be changed to Approved.

 Thank you again for giving me the opportunity to review this work, which I have admired for a long time.

Is the work clearly and accurately presented and does it cite the current literature?

Partly

If applicable, is the statistical analysis and its interpretation appropriate?

Partly

Are all the source data underlying the results available to ensure full reproducibility?

Yes

Is the study design appropriate and is the work technically sound?

Partly

Are the conclusions drawn adequately supported by the results?

Yes

Are sufficient details of methods and analysis provided to allow replication by others?

Yes

Reviewer Expertise:

NA

I confirm that I have read this submission and believe that I have an appropriate level of expertise to confirm that it is of an acceptable scientific standard.

# Peer review: Reviewer response for version 1

**DOI:** 10.21956/wellcomeopenres.17103.r45465

**Published:** 2021-09-14

**Authors:** Erminia Colucci

**Affiliations:** 1 Department of Psychology, Middlesex University, London, UK

Thank you for the opportunity to review this paper. I am familiar with McGuiness'suicide related artworks and was pleased to see they were brought together in this paper, which evaluate its potential use for community-based suicide prevention/intervention initiative. While I see the value of this paper and hope it will be widely read and used, I have a few suggestions:

1. As many international readers will not be familiar with Fort Dunree, I suggest to add or substitute with 'rural Ireland' in the title and/or abstract.
2. Keyword science-arts might be difficult to retrieve so I suggest using the two words separately as well as add creativity and prevention.
3. Unless it is a requirement by Wellcome, I suggest to insert images as picture/jpeg files which will be accessible to anyone instead of only readers with Powerpoint access.
4. The introduction is a bit lengthy and it might be beneficial to shorten it or divide in subsections. Furthermore, while there is some reference to suicide community intervention literature, there is no reference to other scholars and artists who have carried out similar initiatives such as Mic Eales and his Inspired lives and Too Few Ladders works and his arts-based published PhD, which offers also a lived experience perspective that could enrich the Intro and discussion of this paper.
5. The table should be only one with all 5 questions and including Chi square values.
6. Table 2 indicates that the question asked was 'Do you think Lived Lives could somehow benefit people with mental health difficulties?' but the discussion also mention suicidal feelings for this question, please clarify.
7. The discussion is also rather lengthy and some themes could have less number of quotations. Furthermore, a few of the references mentioned seem not to be so relevant for the discussion while some relevant literature such as Eales' or other works that have engaged with creative community-based interventions are not included.
8. One of the main limitations of the study is that there are only 2 basic questions about the potential (positive) impact of the event which seems very little to make any conclusion. Also, the potential negative impact cannot be inferred by lack of mentioning in open ended questions as this question was not expressly asked and I would list this as a limitation. On the other side, the qualitative comment parts of this short questionnaire seems to suggest some potential positive impact which, as the authors mentioned, should be explored more in depth in future studies.

Thank you again and all the best with this and future works!

Is the work clearly and accurately presented and does it cite the current literature?

Partly

If applicable, is the statistical analysis and its interpretation appropriate?

Partly

Are all the source data underlying the results available to ensure full reproducibility?

Yes

Is the study design appropriate and is the work technically sound?

Partly

Are the conclusions drawn adequately supported by the results?

Yes

Are sufficient details of methods and analysis provided to allow replication by others?

Yes

Reviewer Expertise:

arts-based research; visual methods; suicide; suicide prevention; cultural and global mental health

I confirm that I have read this submission and believe that I have an appropriate level of expertise to confirm that it is of an acceptable scientific standard, however I have significant reservations, as outlined above.

# Response

**Published:** 2021-09-28

**Authors:** kevin malone

**Affiliations:** ucd, Ireland

Dear Dr Colucci,

 Thank you for taking the time to review our paper and for your valuable insights and comments.

 Based on your suggestions, we have made a number of changes which we feel have improved the quality of the paper.  We have edited the title and abstract of the paper to clarify the Irish context for international readers. We have contacted Wellcome re updating the keywords and making images accessible in multiple formats. We have edited the introduction, results and discussions sections in an attempt to make them more concise and focused, while also adding the highly relevant work of Mic Eales to our discussion of arts-science literature and other projects with similar aims and themes. In relation to the tables in the results section, we have condensed the previous five tables in to one. This includes the data on experiences of suicidal feelings which was previously presented in table five. Given the number of demographic variables included in the chi-square analyses, it was not possible to include these in the same table without a loss of focus/clarity for the reader although we acknowledge this would have been desirable. In response to your comments on the limitations of the study in terms of potential negative impacts, we have edited the discussion section to acknowledge this important point and discuss potential biases at play.

 We would like to take this opportunity to thank you again for your input and constructive comments.

 Kind Regards,

*Lived Lives *Research Team

# Peer review: Reviewer response for version 1

**DOI:** 10.21956/wellcomeopenres.17103.r43529

**Published:** 2021-05-26

**Authors:** Hyeouk Chris Hahm

**Affiliations:** 1 School of Social Work, Boston University, Boston, MA, USA

Despite the serious and ever-increasing rates of suicide in this world, effective intervention to tackle this complex problem is rare. Lived Lives is an incredibly innovative, bold, and mind-blowing intervention. Even though this study used a small sample size, the implications of the study are critical in the prevention of suicide, especially in the marginalized community. Unlike most suicide interventions, Lived Lives uses community-based approaches to engage and generate support within the community to educate the mental health issues and prevent suicide.

 These are suggestions to improve the paper. 

- The introduction is very informative, but it is rather too long.
- Tables should show %.
- Table 1-Table 5 should be merged to be only one table instead of having each question into each table.
- Please find a way to incorporate the Chi-square analysis into the same table as well.
- The results and discussion sections are also too long. Please cut down about ⅓ lengths. The writing should be more concise. For the Results, the qualitative examples can be more carefully chosen to cut down the lengths. 
- In discussion, I suggest that authors articulate how Lived Lives can be replicated more widely and cross-nationally. 

Is the work clearly and accurately presented and does it cite the current literature?

Yes

If applicable, is the statistical analysis and its interpretation appropriate?

No

Are all the source data underlying the results available to ensure full reproducibility?

Yes

Is the study design appropriate and is the work technically sound?

Yes

Are the conclusions drawn adequately supported by the results?

Yes

Are sufficient details of methods and analysis provided to allow replication by others?

Yes

Reviewer Expertise:

Mental health, suicide prevention.

I confirm that I have read this submission and believe that I have an appropriate level of expertise to confirm that it is of an acceptable scientific standard.

# Response

**Published:** 2021-09-28

**Authors:** kevin malone

**Affiliations:** ucd, Ireland

Dear Dr Hahm,

 Thank you for taking the time to review our paper and for your valuable comments and suggestions.

 Based on your feedback, we have edited the introduction, results and discussions sections in an attempt to make these sections more concise and focused. We have also re-formatted the previous five tables into one, allowing for easier reading of the information including percentages. Given the number of demographic variables included in the chi-square analyses, it was not possible to include these in the same table without a loss of focus/clarity for the reader although we acknowledge this would have been desirable. We have also directed readers to where they can read further discussion of how *Lived Lives *may be replicated more widely.

 We hope you find these changes have improved the quality of our paper and we thank you again for your input.

 Kind Regards,

*Lived Lives *Research Team
